# Retraction: Downregulation of FBP1 Promotes Tumor Metastasis and Indicates Poor Prognosis in Gastric Cancer via Regulating Epithelial-Mesenchymal Transition

**DOI:** 10.1371/journal.pone.0340232

**Published:** 2026-01-05

**Authors:** The PLOS One Editors

Following the publication of this article [1] concerns were raised with results presented in Figs 4 and 5. Specifically,

- The following western blot results appear similar:
  - Figs 4B AGS FBP1 and Fig 5B AGS FBP1 of this article [1], and Fig 6B AGS LDHA of [2], despite presenting different associated loading controls and reporting different aspect ratios.
  - Figs 4B MGC803 FBP1 and Fig 5B MGC80 FBP1, despite presenting different associated loading controls and reporting different aspect ratios.
- The Fig 4F AGS Vector (Migration) panel appears to partially overlap with the Fig 5G AGS Vector (Invasion) panel.
- The Fig 4F MNK45 scr (Migration) and MNK45 shFBP1 (Migration) panels of this article [1] appear similar to the Fig 4 AGS shMMP16 (Invasion) and AGS scramble (Invasion) panels of [3] respectively.
- The MGC-803 cell line reported in this study as a gastric carcinoma cell line has been identified as a contaminated, HeLa cell line instead [5,6].

The authors did not respond to the journal’s request for comments.

In light of the above concerns which call into question the reliability and integrity of the published results, the *PLOS One* Editors retract this article.

All authors either did not respond directly or could not be reached.

In addition to the above concerns, the *PLOS One* Editors are also aware of similarities between:

- The Fig 5B AGS β-actin and Fig 5B MGC803 β-actin of this article [1], and Fig 6B AGS β-actin and Fig 6B MGC803 β-actin of [2].
- The Fig 5D AGS-FBP1 E-cadherin panels of this article [1] and the Fig 5D MGC803 GLUT4 panels of [4].

PLOS notes that [2] and [4] were published at a later date by researchers who do not appear to share affiliations with the authors of [1].
