# Correction: Retraction: Downregulation of FBP1 promotes tumor metastasis and indicates poor prognosis in gastric cancer via regulating epithelial-mesenchymal transition

**DOI:** 10.1371/journal.pone.0341489

**Published:** 2026-01-27

**Authors:** The PLOS One Editors

The second bullet point in the Retraction notice [1] for this article [2] is incorrect. The correct bullet point is:

- The Fig 4F AGS Vector (Migration) panel appears to partially overlap with the Fig 4G AGS Vector (Invasion) panel.

The publisher apologizes for the error.
